# Model Assembly for Estimating Cell Surviving Fraction for Both Targeted and Nontargeted Effects Based on Microdosimetric Probability Densities

**DOI:** 10.1371/journal.pone.0114056

**Published:** 2014-11-26

**Authors:** Tatsuhiko Sato, Nobuyuki Hamada

**Affiliations:** 1 Research Group for Radiation Protection, Nuclear Science and Engineering Center, Japan Atomic Energy Agency (JAEA), Tokai, Ibaraki, Japan; 2 Radiation Safety Research Center, Nuclear Technology Research Laboratory, Central Research Institute of Electric Power Industry (CRIEPI), Komae, Tokyo, Japan; University of Pittsburgh School of Medicine, United States of America

## Abstract

We here propose a new model assembly for estimating the surviving fraction of cells irradiated with various types of ionizing radiation, considering both targeted and nontargeted effects in the same framework. The probability densities of specific energies in two scales, which are the cell nucleus and its substructure called a domain, were employed as the physical index for characterizing the radiation fields. In the model assembly, our previously established double stochastic microdosimetric kinetic (DSMK) model was used to express the targeted effect, whereas a newly developed model was used to express the nontargeted effect. The radioresistance caused by overexpression of anti-apoptotic protein Bcl-2 known to frequently occur in human cancer was also considered by introducing the concept of the adaptive response in the DSMK model. The accuracy of the model assembly was examined by comparing the computationally and experimentally determined surviving fraction of Bcl-2 cells (Bcl-2 overexpressing HeLa cells) and Neo cells (neomycin resistant gene-expressing HeLa cells) irradiated with microbeam or broadbeam of energetic heavy ions, as well as the WI-38 normal human fibroblasts irradiated with X-ray microbeam. The model assembly reproduced very well the experimentally determined surviving fraction over a wide range of dose and linear energy transfer (LET) values. Our newly established model assembly will be worth being incorporated into treatment planning systems for heavy-ion therapy, brachytherapy, and boron neutron capture therapy, given critical roles of the frequent Bcl-2 overexpression and the nontargeted effect in estimating therapeutic outcomes and harmful effects of such advanced therapeutic modalities.

## Introduction

Systematic investigation of cell survival is of great importance in the treatment planning of heavy-ion therapy as well as to better understand the mechanism for its high relative biological effectiveness (RBE) compared with conventional photon therapy. A series of biological studies have determined the clonogenic survival of various cell types irradiated with different types of energetic heavy ions [1]. Several models were developed to reproduce such experimentally determined data [2]–[9], some of which have been implemented into the treatment planning system for heavy-ion therapy [5], [6].

Heavy-ion therapy is effective at inactivating photon-resistant tumors [10], [11]. For instance, an anti-apoptotic factor Bcl-2 is overexpressed in the tumors of 35–50% of cancer patients [12], but heavy-ion irradiation can overcome tumor radioresistance caused by such Bcl-2 overexpression [13], [14]. However, its underlying mechanisms remain incompletely understood, and there is no model available that can explicitly consider such “Bcl-2 effect” in estimating cell survival. Establishment of such model should improve accuracy of predicting outcomes of heavy-ion therapy.

In addition to targeted effects that occur in nucleus-irradiated cells, there is convincing evidence that heavy-ion irradiation can cause nontargeted effects in bystander cells that have not themselves been irradiated but received signals from irradiated cells [15]–[18]. Nontargeted effects are important not only in estimating harmful effects on normal tissues outsider the target volume in heavy-ion therapy, but also in estimating effects of brachytherapy and boron neutron capture therapy (BNCT) because non-irradiated cells coexist with irradiated cells within the target volume [19]. Several studies have been devoted to developing models for quantitative description of cell survival considering nontargeted effects [20]–[26], but most of them are only limitedly applicable to idealized irradiation conditions (e.g. microbeam irradiation, split-field irradiation, or medium-transfer experiments). The radiation fields in patients generally consist of various particles with a wide range of energy, and development of a new model applicable to such complex radiation fields is hence necessary to consider the nontargeted effect in the treatment planning.

From these considerations, we here set out to develop a new model assembly for estimating cell survival related to targeted and nontargeted effects in the same framework. Instead of the mean absorbed dose and linear energy transfer (LET) values, the probability density (PD) of specific energy in microscopic sites [27] was employed as the physical index for characterizing the radiation fields in order to represent the dose inhomogeneity in both microbeam and broadbeam irradiation experiments. The targeted effect was expressed by our previously developed double stochastic microdosimetric kinetic (DSMK) model [28], which can estimate cell surviving fraction (SF) in any radiation fields according to the number and localization of lesions created in a cell nucleus. We here further improve the DSMK model to be capable of explicitly describing cell inactivation related to the Bcl-2 effect. The nontargeted effect was expressed by improving our original model [29], which assumed that a cell is potentially inactivated when receiving an apoptotic signal from irradiated cells.

In this study, the parameters used in the model assembly were determined by the least-square (LSq) fitting of the experimentally determined SF of Bcl-2 cells (Bcl-2 overexpressing HeLa cells) and Neo cells (neomycin resistant gene-expressing HeLa cells) irradiated with microbeam or broadbeam of heavy ions [13], [16], as well as the WI-38 normal human fibroblasts irradiated with X-ray microbeam [30]. The details of the calculation procedures together with the comparison results between the computationally and experimentally determined SF are given below.

## Theory and Calculation

In the development of the model assembly, we assumed that the SF related to the targeted and nontargeted effects (expressed as *S*_T_ and *S*_NT_, respectively) can be calculated separately, and that the total cell survival fraction, *S*, can be written by their product: namely
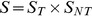
(1)


It should be mentioned that potential contribution of the synergetic effect between the targeted and nontargeted effects was considered as a correction factor of *S*_NT_ as described later.

The targeted effect was further divided into two components. One is for the Bcl-2 effect that induces extra inactivation of cells that do not overexpress Bcl-2, and the other is for targeted effects other than the Bcl-2 effect (hereafter referred to as the conventional targeted effects). We assumed that different types of lesions created in a cell nucleus independently trigger cell inactivation related to each of these two types of effects. Considering that the Bcl-2 effect was evident at low LET but not at high LET, we introduced the following equation to express *S*_T_:
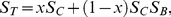
(2)where *S*_B_ and *S*_C_ are the SF related to the Bcl-2 effect and the conventional targeted effect, respectively. Irrespective of *x* (the ratio of Bcl-2 overexpressing cells), the value of *S*_T_ becomes closer to *S*_C_ with an increase in LET when *S*_B_ asymptotes to 1 for high-LET irradiation. In our model assembly, the DSMK model was used to calculate these SF.

### Estimation of Surviving Fraction Related to Targeted Effect

#### Principle of the DSMK Model

The DSMK model was developed on the basis of the microdosimetric kinetic (MK) model proposed by Hawkins [2], [8]. In both DSMK and MK models, the following basic assumptions were made: (*i*) a cell nucleus can be divided into a number of microscopic sites called domains; (*ii*) radiation exposure produces two types of DNA damage named lethal and sublethal lesions in cell nuclei; (*iii*) the specific energy *z* in the domain determines the number of lethal and sublethal intra-domain lesions; (*iv*) a sublethal lesion is to be repaired or converted into a lethal lesion via spontaneous transformation or interaction with another sublethal lesion created in the same domain; (*v*) a domain is to be considered inactivated when an intra-domain lethal lesion is formed; and (*vi*) a cell is to be considered inactivated when inactivation of an intranuclear domain took place.

The most important difference between the DSMK and MK models is that the DSMK model fully considers the stochastic nature of both domain and cell-nucleus specific energies, while the MK model represents the stochastic nature by their approximated mean values and variances. Besides, the DSMK model considers the saturation in the production rates of lethal and sublethal lesions per specific energy *z* in a domain for expressing an overkill effect of high-LET irradiation. Owing to these profiles, the DSMK model can reproduce the experimentally determined SF for high-LET and high-dose irradiation [31], whereas the original MK model tends to underestimate the data. Calculation procedures for the DSMK model are outlined below, details of which have been previously described [28].

In the DSMK model, the SF of a single cell with its nucleus specific energy *z*_n_ can be calculated by
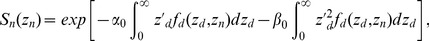
(3)where *f*_d_(*z*_d_,*z*_n_) denotes the PD of domain specific energy, *z*_d_, for *z*_n_, and it holds:



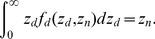
(4)The parameters α_0_ expresses the inactivation sensitivity of domains to lethal lesions, and β_0_ represents the interaction probability of two sublethal lesions created in the same domain, where their numerical values are independent of the radiation imparting the energy. The parameter 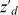 denotes the saturation-corrected specific energy, which is an effective quantity proportional to the lesions produced in a domain. In a similar manner as given in the International Commission on Radiation Units and Measurements Report 36 [27], we assumed that 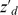 can be calculated by
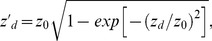
(5)where *z*_0_ is the saturation parameter. The SF related to the targeted effect for a cell group irradiated with absorbed dose *D*, *S*_T_(*D*), can be estimated by
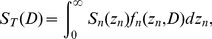
(6)where *f*_n_(*z*_n_,*D*) is the PD of *z*_n_ for absorbed dose *D*, which can be written as



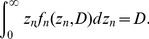
(7)Eqs. (3) and (6) are the fundamental equations that need to be solved in the DSMK model. For this purpose, the numerical values of α_0_, β_0_, and *z*_0_, together with the domain and cell-nucleus radii, *r*_d_ and *r*_n_, respectively, are necessary to be determined.

#### Parameter Determination for the DSMK Model

In this study, the DSMK model parameters for the conventional targeted effect and the Bcl-2 effect were separately determined from the SF of Bcl-2 cells and Neo cells irradiated with broadbeam of photons or heavy ions [13]. The origin of these two cell lines is HeLa, but Bcl-2 overexpression occurs only in Bcl-2 cells [32]–[34]. Assuming that the value of *x* in Eq. (2) equals 1 and 0 for Bcl-2 and Neo cells, respectively, the SF related to the Bcl-2 effect can be experimentally derived by
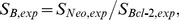
(8)where *S*_Neo,exp_ and *S*_Bcl-2,exp_ are the experimentally determined SF for Neo cells and Bcl-2 cells, respectively, for the same irradiation condition. On the other hand, the SF related to the conventional targeted effect cannot be directly obtained from experiments because contributions of the targeted effects and nontargeted effects are indistinguishable. We therefore estimated the experimentally determined SF related to the conventional targeted effect, *S*_C,exp_, from the experimentally determined SF for the Bcl-2 cells by computationally excluding the contribution from nontargeted effect as follows:
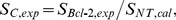
(9)where *S*_NT,cal_ is the SF related to the nontargeted effect calculated by Eq. (16), as described later in this paper. Note that *S*_Bcl-2,exp_ is generally much smaller than *S*_NT,cal_ except at a low dose, and the influence of this correction in the LSq fitting is thus not so significant.

Numerical values of *f*_d_(*z*_d_,*z*_n_) and *f*_n_(*z*_n_,*D*) for each experimental irradiation condition were calculated using the microdosimetric function and LET-estimator function, respectively, implemented in the Particle and Heavy Ion Transport code System (PHITS) [35]. The microdosimetric function was developed based on the results of track structure simulation [36], which enables the calculation of the PDs of *z* for microscopic sites distributed in macroscopic matter within a reasonable computational time while considering its stochastic nature properly [37], [38]. However, the microdosimetric function and the LET-estimator function in PHITS can calculate the PDs only for the single event distribution. Thus, the influence of the multiple hits on the PDs was considered by iteratively solving the convolution of the PHITS results, or using the database developed by a Monte Carlo method [28].

By substituting the calculated *f*_d_(*z*_d_,*z*_n_) and *f*_n_(*z*_n_,*D*) into Eq. (3) and Eq. (6), respectively, the numerical values of α_0_, β_0_, *z*_0_, and *r*_d_ for the conventional targeted effect and the Bcl-2 effect were separately determined on the basis of the LSq fitting by minimizing the chi-square value, *χ* ^2^, which were calculated by
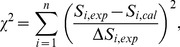
(10)where *S_i_*_,exp_ is the experimentally determined SF for the irradiation condition *i* obtained from Eq. (8) or Eq. (9), Δ*S_i_*_,exp_ is the uncertainties of the experimental data, *S_i_*_,cal_ is the corresponding SF calculated by the DSMK model, and *n* is the number of irradiation conditions, i.e. experimental data points adopted in the fitting. The asymptotic standard errors of each fitting parameter were also estimated by calculating the parameter covariance matrix. The best fit value of *r*_n_ was separately determined by performing the LSq fitting several times by manually changing the parameter, because *r*_n_ should be the same for both the conventional targeted effect and the Bcl-2 effect. Thus, the uncertainty of *r*_n_ was not estimated in this study.

#### Extension of the DSMK Model for the Bcl-2 Effect

The Bcl-2 effect was most evident in the photon exposure, and became less significant at higher LET [13]; namely, the SF related to the Bcl-2 effect was positively correlated with LET even at low LET. In the DSMK model, this tendency can be expressed by the saturation of the number of created lesions related to the Bcl-2 effect per dose, as written by Eq. (5). However, this equation was originally introduced to express the overkill effect of high-LET irradiation – generally at over 100-200 keV/µm, where the number of lesions related to cell killing (e.g. complex DNA damage) per dose tends to decrease with increasing LET [39]. Thus, Eq. (5) is biologically not suitable for representing the positive correlation between the SF and LET observed at low LET, though it is mathematically adequate to express the tendency by setting a very low value to the saturation parameter, *z*_0_, as discussed later in this paper.

From these considerations, we additionally propose an alternative method for calculating the SF related to the Bcl-2 effect by introducing the following hypotheses: (*i*) cell inactivation related to the Bcl-2 effect is induced by a certain type of DNA damage, which triggers apoptosis that can be inhibited by Bcl-2; (*ii*) Bcl-2 works only in domains where repair of such DNA damage has not occurred; and (*iii*) the numbers of the intra-domain DNA damage created and repaired are proportional to its specific energy. The item (*ii*) was introduced based on the concept of the adaptive response [40], where low-dose irradiation renders cells radioresistant by triggering a certain protective mechanism.

Under these hypotheses, the parameter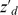, which is proportional to the number of created lesions related to the Bcl-2 effect per domain, can be calculated by
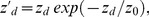
(11)where *z*_0_ in this equation denotes the mean specific energy necessary for inducing DNA repair that disables Bcl-2 function. Replacing Eq. (5) by Eq. (11), the SF related to the Bcl-2 effect can be estimated in an alternative way. The DSMK model employing Eq. (5) and Eq. (11) for determining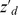 is herein called “saturation-corrected” and “adaptive-response” methods, respectively. For both methods, the LSq fitting was used to separately determine numerical values of not only *z*_0_ but also α_0_, β_0_, and *r*_d_.

### Estimation of Surviving Fraction Related to Nontargeted Effect

#### Principle of the Nontargeted Effect Model

In our nontargeted effect model, the radiation fields were also characterized by *f*_n_(*z*_n_,*D*), which can be utilized in both microbeam and broadbeam irradiation experiments. The fundamental concept of this model was developed on the basis of the Fakir model [23], details of which have been described [29].

The following basic hypotheses were employed in this model: (*i*) the nontargeted effect potentially inactivates a cell upon receipt of an apoptotic signal; (*ii*) apoptotic signals are emitted by irradiated cells triggered with a probability that depends on irradiation conditions, but signal intensity is independent of these conditions; (*iii*) the trigger probability that a cell is turned into a signal-emitting cell after irradiation with its nucleus specific energy *z*_n_, *P*_T_(*z*_n_), can be expressed by
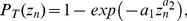
(12)where *a*_1_ and *a*_2_ are parameters that depend on radiation types and cell lines; (*iv*) apoptotic signals uniformly propagate over a certain distance; (*v*) the fraction of cells receiving an apoptotic signal from a single signal-emitting cell is constant; and (*vi*) all cells within the propagation distance can receive an apoptotic signal, but only a certain fraction of cells with *z*_n_ less than a threshold level are actually inactivated. The item (*iii*) was revised from our previous study, while the item (*vi*) was newly introduced in this study.

Under these hypotheses, the fraction of signal-emitting cells in the radiation field with the mean absorbed dose *D*, *P*_S_(*D*), can be calculated by
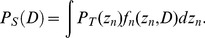
(13)


Numerical values of *f*_n_(*z*_n_,*D*) for broadbeam irradiation experiments were determined using the LET-estimator function of PHITS in combination with the convolution or database method as aforedescribed, whereas those for microbeam irradiation experiments were calculated by
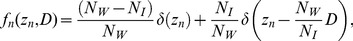
(14)where *N*_I_ is the number of irradiated cells, *N*_W_ is the number of cells in the whole population, and *δ* is the Dirac's delta function. In microbeam irradiation experiments, the mean absorbed dose *D* in the whole cell system is generally not given. In that case, *N*_W_ *D*/*N*_I_ should be replaced by *D*_I_, which is the mean absorbed dose of irradiated cells. Note that the stochastic nature of nucleus specific energies among irradiated cells is not considered in Eq. (14), because the divergence of each nucleus specific energies from their mean value is generally not so large in microbeam experiments owing to the controlled number of particles delivered to each cell.

Assuming that apoptotic signals can propagate throughout the whole cell population, the probability that a cell escapes from reception of the entire signal emitted, *P*_E_(*D*), is then given by the sum of the binomial probabilities, which is written as
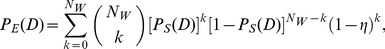
(15)where *η* is the fraction of cells receiving an apoptotic signal from a signal-emitting cell. According to Ref. [23], the calculation of this sum yields 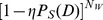. Then, the SF related to the nontargeted effect, *S*_NT_(*D*) can be determined by
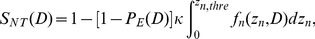
(16)where *κ* is the fraction of non-irradiated cells that are actually inactivated when receiving an apoptotic signal, and *z*_n,thre_ is the threshold cell-nucleus specific energy above which cells become insensitive to the apoptotic signal. Note that the value calculated by the integral in Eq. (16) can be regarded as a correction factor due to the potential synergetic effect between targeted and nontargeted effects. In order to numerically solve Eq. (16), the values of *a*_1_, *a*_2_, *η*, *κ*, and *z*_n,thre_ are necessary to be determined.

#### Parameter Determination for the Nontargeted Effect Model

In this study, we determined the nontargeted model parameters except for *z*_n,thre_ by the LSq fitting of the SF of Bcl-2 cells and Neo cells irradiated with heavy-ion microbeam [16] as well as that of the WI-38 normal human fibroblasts irradiated with synchrotron X-ray microbeam [30]. In these experiments, the number of Bcl-2 cells, Neo cells and WI-38 cells in each whole population, *N*_W_, was 2.67e6, 2.68e6 and 7.0e5, respectively, and such cell population sizes were determined by the conventional automatic counting of trypsinized cells. Note that at >1.9 Gy, the experimentally determined SF of WI-38 cells became higher with increasing dose, but our model failed to reproduce this tendency. Thus, such high-dose irradiation data were excluded in the LSq fitting.

In the LSq fitting, we approximated that numerical values of the parameters were the same for Bcl-2 cells and Neo cells, because the nontargeted effect occurs independent of Bcl-2 overexpression [16]. We also assumed that the parameters related to the dose dependence of the triggering probability, *a*_1_ and *a*_2_, were the same for all cell lines and radiation types, and their numerical values were determined from the X-ray microbeam irradiation experiment. This assumption obviously contradicts the abovementioned hypothesis (*iii*) that defines Eq. (12), but must be introduced in this study because the SF was not experimentally determined as a function of dose in heavy-ion microbeam irradiation experiments. Thus, we evaluated the parameters for WI-38 cells first, then determined those for Bcl2/Neo cells at the fixed *a*_1_ and *a*_2._ Note that these LSq fittings were performed prior to those for the targeted effect model, because the calculated SF related to the nontargeted effect was used in the LSq fitting for the targeted effect model as described in Eq. (9).

It should be mentioned that the numerical value of *z*_n,thre_ cannot be evaluated from such microbeam irradiation experiments, because it affects the SF only for broadbeam irradiation. Thus, we set *z*_n,thre_ to 1 mGy or infinity, and analyzed its influence on the SF for broadbeam irradiation experiments as described below. The condition of *z*_n,thre_ = 1 mGy represents the situation where a cell activates a certain self-protective mechanism against the nontargeted effect when it is directly irradiated as suggested in [41], whereas such a mechanism does not exist in the condition of *z*_n,thre_ → ∞.

### Estimation of RBE-Weighted Doses

The RBE-weighted dose (i.e. the product of the absorbed dose and RBE) is employed in the treatment planning of heavy-ion therapy for expressing its therapeutic efficacy compared with that of photon therapy. To investigate the importance of the consideration of the Bcl-2 effect and the nontargeted effect in the treatment planning, we calculated the RBE-weighted doses in a water slab phantom irradiated by mono-energetic and spread-out Bragg peak (SOBP) carbon-ion beams with or without considering those two effects. For that purpose, PHITS simulations were performed to calculate the PD of domain specific energy, the frequency distributions of LET, and the absorbed doses in a thick water slab phantom irradiated with carbon-ion beams. The primary ion energies were set to 50 and 290 MeV/u. For the 290 MeV/u beam, a ridge filter designed to achieve a constant RBE-weighted dose region over a width of 6 g/cm^2^ [42] was mounted at the upstream of the water phantom. The decrease in beam energy and the nuclear interactions that occurred in the air as well as in the ridge filter were also taken into account in the simulation. These simulation setups were the same as those employed in our previous study [28].

The calculated absorbed doses were normalized to 2 Gy at the Bragg peak and at the center of SOBP for 50 and 290 MeV/u beams, respectively. The SF for cell groups with different Bcl-2 overexpression status, i.e. *x* = 0, 0.5, and 1 in Eq. (2), was respectively calculated as a function of the depth from the front surface of the water phantom using our developed model assembly with or without considering the nontargeted effect. Note that *x* = 0 and 1 represents the cell groups solely comprising Neo and Bcl-2 cells, respectively, while *x* = 0.5 represents the situation that Neo and Bcl-2 cells coexist with the same density. The calculated SF at each depth was then converted into the RBE-weighted dose by finding the corresponding photon dose that gives the same SF. For this conversion, the SF for the irradiation of γ-rays from ^6^°Co was also calculated as the reference dose-response curves, using our developed model assembly.

## Results and Discussion

### Evaluated Parameters

Tables 1 and 2 summarize the evaluated parameters and their asymptotic standard errors for the nontargeted and targeted effect models, respectively, together with the chi-square values per the degrees of freedom, *χ* ^2^/df, obtained from the LSq fitting. The 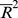 values were also given in the tables, which can be calculated by
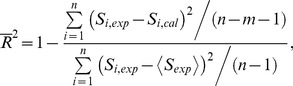
(17)where *m* is the number of the fitting parameters, 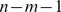 indicates the degrees of freedom, and 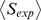 represents the mean value of the experimentally determined SF. Note that 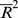 is an adjustment for the coefficient of determination, *R* ^2^, which indicates how a model fits data well.

**Table 1:** Evaluated parameters for the nontargeted effect model for WI-38 and Bcl-2/Neo cells.

	WI-38		Bcl-2/Neo	
*a*_1_	18500	(6.50E+7)	18500	
*a*_2_	5.38	(1530)	5.38	
*η*	0.604	(0.523)	0.0596	(0.0413)
*κ*	0.106	(0.0141)	0.147	(0.0251)
*χ* ^2^/df	0.943		0.468	
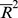	0.467		0.265	

The values in the parentheses are the asymptotic standard errors; The parameters *a*_1_ and *a*_2_ were evaluated by giving *z*_n_ in Gy in Eq.(12).

**Table 2:** Evaluated parameters for the targeted effect model for Bcl-2/Neo cells.

	Conventional Targeted Effect				Bcl-2 Effect			
	zn,thre = 1 mGy		zn,thre → ∞		Saturation-Correction		Adaptive-Response	
α0 (Gy-1)	−0.0707	(0.101)	−0.0596	(0.0883)	0.454	(0.290)	0.493	(0.328)
β0 (Gy-2)	0.00325	(0.00350)	0.00413	(0.00309)	0		0	
rd (µm)	0.0562	(0.0392)	0.0654	(0.0204)	0.300	(0.191)	0.349	(0.197)
z0 (Gy)	1340	(86.5)	993	(60.1)	2.51	(3.50)	6.51	(10.1)
rn (µm)	7.8		7.8		7.8		7.8	
χ2/df	0.910		0.779		0.340		0.314	
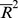	0.961		0.941		0.497		0.544	

The values in the parentheses are the asymptotic standard errors.

**Figure 1:**
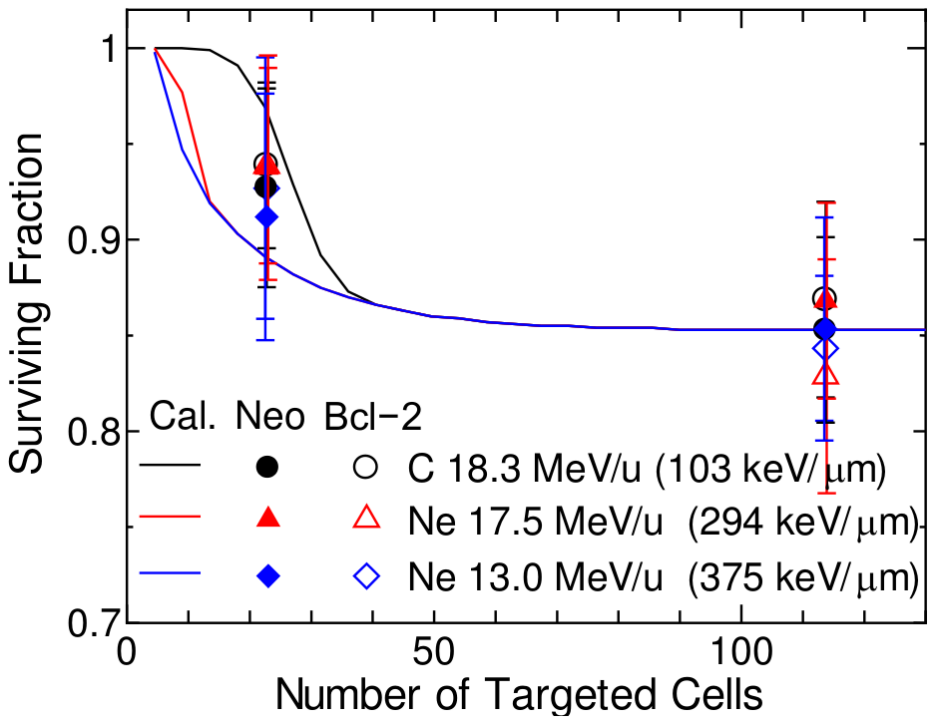
Surviving fraction of Bcl-2 cells and Neo cells irradiated with heavy-ion microbeam. Lines indicate the data obtained from our nontargeted effect model with its parameters given in Table 1, whereas solid and open symbols indicate the experimentally determined data for Neo cells and Bcl-2 cells, respectively, taken from Ref. [16]. The number of particles passing through each targeted cell was fixed to 10. LET of the primary ions is shown in parentheses.

**Figure 2:**
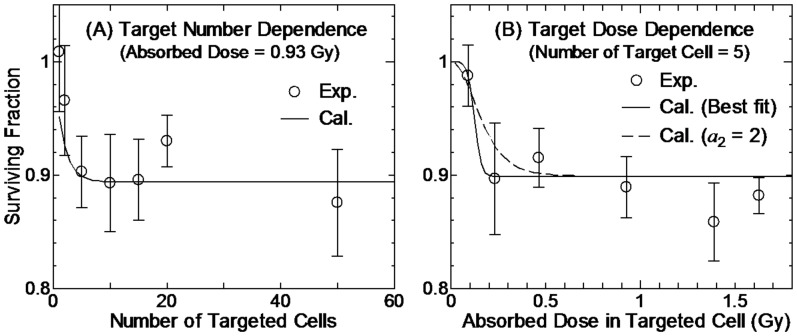
Surviving fraction of WI-38 cells irradiated with synchrotron X-ray microbeam. Panels A and B show the data as a function of the number of targeted cells and their absorbed doses, respectively. The absorbed dose in each targeted cell was 0.93 Gy in Panel A, and the number of targeted cells was 5 in Panel B. Solid lines indicate the data obtained from our nontargeted effect model with its parameters given in Table 1, whereas dash line denotes the data obtained by fixing *a*_2_ = 2. Open circles indicate the experimentally determined data taken from Ref. [30].

The 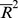
values for the conventional targeted effect were very close to 1, indicating the accuracy of the DSMK model. On the other hand, the 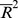
values for the nontargeted effect model were rather small because the variances of the experimentally determined SF related to the nontargeted effect were not so large, i.e. the SF was within the range between 0.8 and 1.0 as shown in Figures 1 and 2.

For the conventional targeted effect, we respectively determined two sets of the parameters for *z*_n,thre_ = 1 mGy and *z*_n,thre_ → ∞, because the experimentally determined SF related to the conventional targeted effect, *S*_C,exp_, used in the LSq fitting depends on the computationally determined SF related to the nontargeted effect, *S*_NT,cal_, as written by Eq. (9). Two sets of the parameters were also given for the Bcl-2 effect: one was for the saturation-corrected method and the other was for the adaptive-response method.

The numerical value and the standard error of the β_0_ parameter for the Bcl-2 effect were fixed to 0 in the LSq fitting; otherwise, it became a negative value. It should be mentioned that β_0_ represents the interaction probability of two lesions created in the same domain, and that β_0_ = 0 thus suggests that the lesions triggering the Bcl-2 effect do not interact with one another. Therefore, the domain radius, *r*_d_, for the Bcl-2 effect is independent of the diffusible distance of the lesions, and its numerical values are largely different from those for the conventional targeted effect.

Tables 3 and 4 show examples of the covariance matrixes of the evaluated parameters. The asymptotic standard errors given in Tables 1 and 2 were determined from the square root of the diagonal elements of the covariance matrixes. It is evident from the tables that the uncertainties of the evaluated parameters were generally large, especially for *a*_1_ and *a*_2_ used in the nontargeted model. This tendency results from the insufficient experimental data sets for various irradiation conditions as well as the large uncertainties of the available experimental data. Thus, further investigation is needed to more precisely determine the fitting parameters for deducing biological profiles from the evaluated parameters.

**Table 3:** Covariance matrix of the evaluated parameters for the nontargeted effect model for the WI-38 cells.

	*a*_1_	*a*_2_	*η*	*κ*
*a*_1_	4.22E+15	9.97E+10	−2.18E+06	−60400
*a*_2_	9.97E+10	2.35E+06	−51.4	−1.43
*η*	−2.18E+06	−51.4	0.274	−0.00556
*κ*	−60400	−1.43	−0.00556	0.000198

The parameters *a*_1_ and *a*_2_ were evaluated by giving *z*_n_ in Gy in Eq.(12).

**Table 4:** Covariance matrix of the evaluated parameters for the conventional targeted effect model for *z*_n,thre_ → ∞.

	*α*_0_ (Gy^−1^)	*β*_0_ (Gy^−2^)	*r*_d_ (µm)	*z*_0_ (Gy)
*α*_0_ (Gy^−1^)	0.00780	0.000265	0.00176	4.93
*β*_0_ (Gy^−2^)	0.000265	0.00000958	0.0000630	0.161
*r*_d_ (µm)	0.00176	0.0000630	0.000416	1.09
*z*_0_ (Gy)	4.93	0.161	1.09	3620

### Surviving Fraction for Microbeam Irradiation Experiments

The computationally and experimentally determined SF for the microbeam irradiation experiments [16], [30] are compared in Figures 1 and 2. In the model calculation, we assumed that the SF related to the targeted effect was 1 for all irradiation conditions, because the fraction of targeted cells among the whole cell population was negligibly small (<0.01%). It is evident from Figures 1 and 2 that our nontargeted effect model can reproduce the experimental data fairly well, though the experimental uncertainties were so large that the underlying hypotheses introduced in our nontargeted effect model still need to be validated in further studies.

Figure 1 indicates that the computational determined SF for three types of heavy ions was different from one another in spite of the same model parameters for all radiations. This was simply because absorbed dose in each targeted cell varies with LET of the incident particles at the fixed number of particles delivered to each cell.

Panel B in Figure 2 illustrates that the experimentally determined SF steeply fell off at around 0.2 Gy. Thus, the best fit value of the *a*_2_ parameter in Eq. (12) was as high as 5.38. This is the reason why we introduced a free power index of *z*_n_ for expressing the triggering probability of the non-target effect in this study, instead of the linear quadratic relation employed in our previous work [29]. However, it is difficult to judge which equation is more appropriate for expressing the triggering probability only from the available experimental data, because the *a*_2_ value had little influence on the computationally determined SF, and because its standard error was very large. For example, Panel B of Figure 2 also depicts the computationally determined SF obtained by fixing *a*_2_ = 2 in the LSq fitting. The data for the fixed *a*_2_ seem to reproduce the experimentally determined SF in equivalent to the data for the best fit value; the *χ* ^2^ value was increased by only approximately 4% owing to the constant *a*_2_. This necessitates further studies to investigate the dose dependence of triggering the nontargeted effect.

### Surviving Fraction for Broadbeam Irradiation Experiments

**Figure 3:**
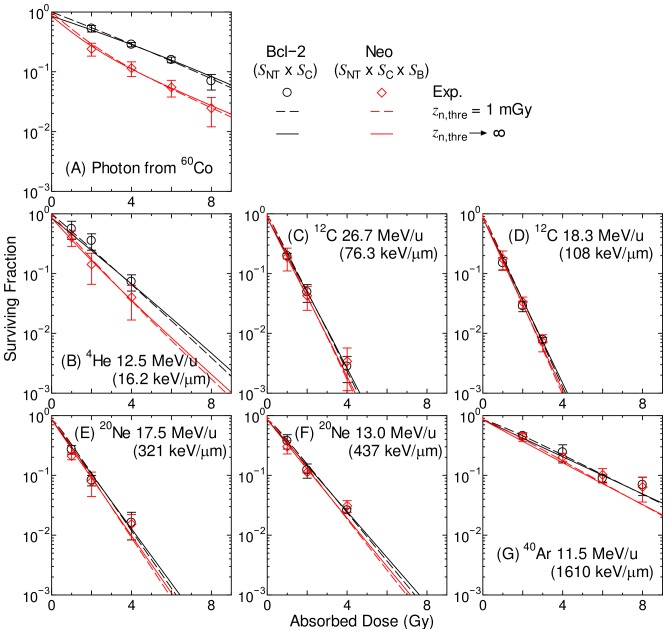
Surviving fraction of broadbeam-irradiated Bcl-2 cells and Neo cells. The calculated data for Bcl-2 cells and Neo cells were obtained from *S*_NT_ x *S*_C_ and *S*_NT_ x *S*_C_ x *S*_B_, respectively, where *S*_NT_, *S*_C_, and *S*_B_ are the SF related to the nontargeted effect, conventional targeted effect, and Bcl-2 effects, respectively. The experimental data were taken from [13]. LET of the primary ions is shown in parentheses.

Figure 3 shows the computationally determined SF of broadbeam-irradiated Bcl-2 cells and Neo cells, in comparison to the corresponding experimental data [13]. The computationally determined data for Neo cells were obtained by employing the adaptive-response method instead of the saturation-correction method, and the difference between these two methods will be discussed later in this section. Excellent agreements were observed between the computationally and experimentally determined SF for all irradiation conditions, indicating the accuracy of the model assembly established in this study. In the calculation, the nontargeted effect was also considered by setting *z*_n,thre_ to 1 mGy or infinity, though the results seems to be almost independent of *z*_n,thre_ in Figure 3. This is because the nontargeted effect plays an important role only at low dose in our model assembly. Note that SF for photon from ^60^Co shown in the panel (A) of Fig. 3 was employed as the reference dose-response curves in the RBE-weighted dose estimation.

**Figure 4:**
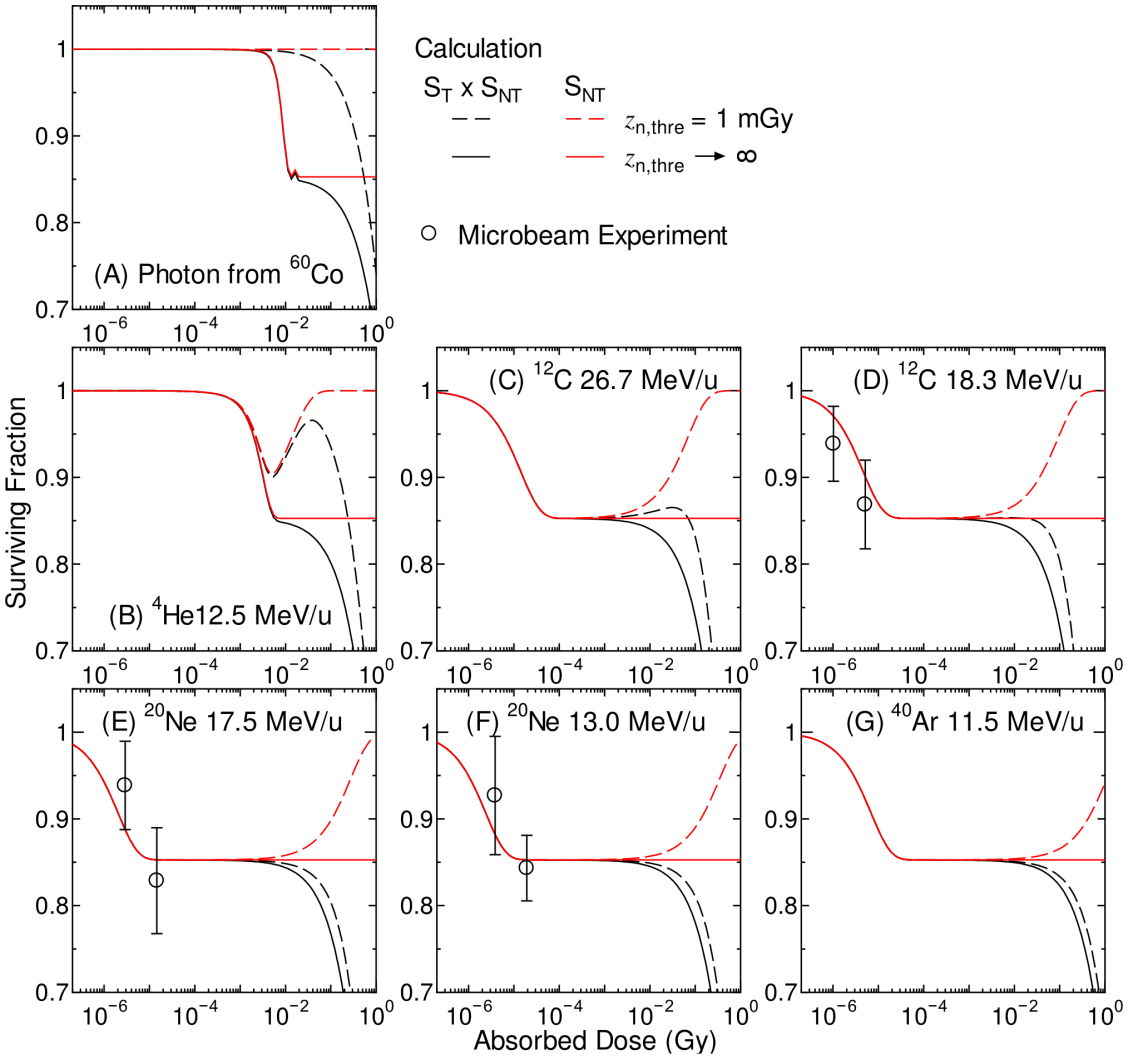
Surviving fraction of the Bcl-2 cells irradiated with various broadbeams below 1 Gy. Black and red lines indicate the computationally determined SF with or without considering the targeted effect, respectively. The data obtained from the microbeam experiments [16] are also plotted in the graphs.

Figure 4 shows the computationally determined SF of Bcl-2 cells below 1 Gy, together with the data from microbeam irradiation experiments [16] for which absorbed dose was simply estimated from the mean absorbed dose of irradiated cells multiplied by the fraction of irradiated cells among the whole cell population. The computationally determined SF only related to the nontargeted effect is also plotted in Figure 4.

It is evident from Figure 4 that the calculated SF very well agrees with the experimental data, indicating that our established model assembly can reproduce the cell SF irrespective of the irradiation conditions such as dose levels and beam profiles. When *z*_n,thre_ was set to 1 mGy, the non-target effect became less significant with the increase of absorbed dose above the threshold, because most cells became unresponsive to apoptotic signals. Thus, dose response of the computationally determined SF exhibited low-dose hypersensitivity [43], [44]. The absorbed dose giving the minimum SF related to the hypersensitivity obtained in this study was smaller than those generally observed – around 0.3 Gy. However, this value can be adjusted by changing the model parameters such as *a*_2_ in Eq. (12) and *z*_n,thre_ whose numerical values have not been precisely evaluated. Thus, our model has a potential to reproduce the SF of cells that show low-dose hypersensitivity.

**Figure 5:**
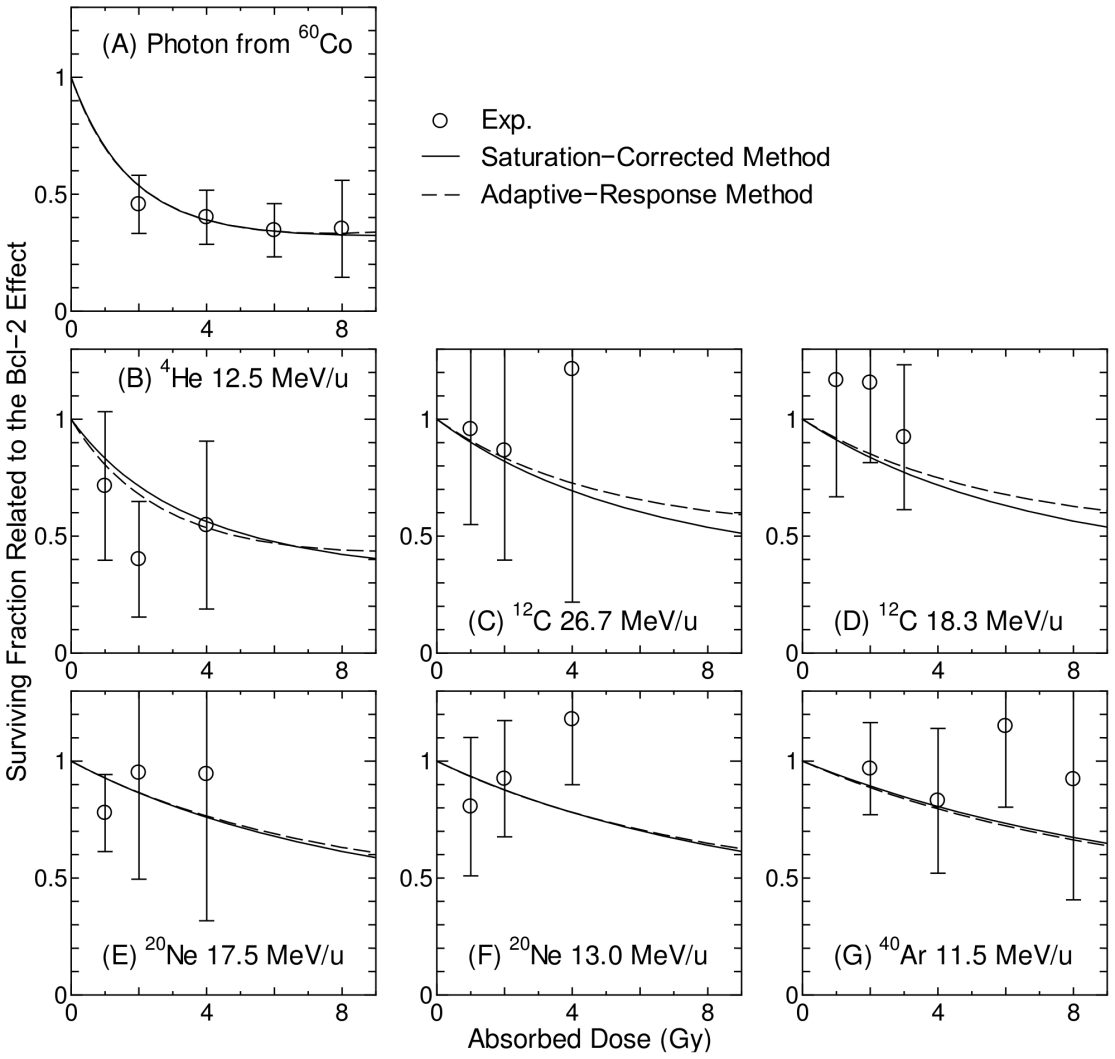
Surviving fraction related to the Bcl-2 effect calculated by the saturation-corrected and adaptive-response methods. The corresponding experimental data [13] obtained from Eq. (8) are also plotted.

Figure 5 shows the SF related to the Bcl-2 effect calculated by the saturation-corrected and adaptive-response methods, in comparison to the corresponding experimental data obtained from Eq. (8). It was found from Figure 5 that the SF increases with increasing LET, and that both calculation methods can reproduce the tendency fairly well. Albeit no significant difference between these two calculation methods, the adaptive-response method can reproduce the experimental data for the photon irradiations in a better precision. Thus, the 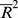 value for the adaptive-response method was greater than that for the saturation-corrected method (Table 2). Considering the larger 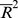 value and the biologically reasonable assumptions as discussed in the previous section, the adaptive response method would be more reliable than the saturation-corrected method for estimating the SF related to the Bcl-2 effect. Note that the evaluated *z*_0_ value for the saturation-correction method, 2.51 Gy, corresponds to the lineal energy of 4.44 keV/µm, which is generally considered to be too small to cause the saturation of DNA damage.

### RBE-Weighted Doses

**Figure 6:**
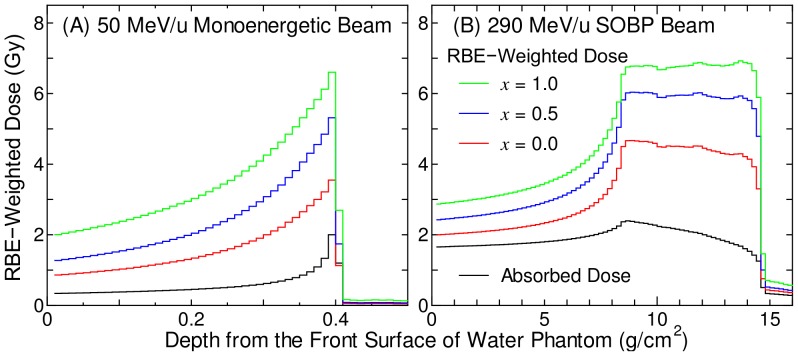
Calculated RBE-weighted doses and absorbed doses in water phantom irradiated by carbon beams. Panels A and B show the data for 50 MeV/u mono-energetic and 290 MeV/u spread-out Bragg peak (SOBP) beams, respectively. The RBE-weighted doses were calculated for cell groups with different Bcl-2 overexpression status, i.e. *x* = 0, 0.5, and 1 in Eq. (2). The nontargeted effect was considered in the calculation, although it caused little impact on the RBE calculation for such high-dose irradiations.

Figure 6 shows the calculated RBE-weighted doses and absorbed doses as a function of the depth from the front surface of the water phantom irradiated with 50 MeV/u mono-energetic and 290 MeV/u SOBP carbon-ion beams. The parameters listed in the righter columns of Tables 1 and 2, i.e. the Bcl-2/Neo cells, *z*_n,thre_ → ∞, and the adaptive-response method for the nontargeted effect, the conventional targeted effect, and the Bcl-2 effect, respectively, were employed in the RBE estimation. The statistical uncertainties of these calculated data were enough small, i.e. less than 1% in most cases.

It should be mentioned that the RBE-weighted doses calculated with or without considering the nontargeted effect were almost identical to each other, although the data obtained without considering the effect were not depicted in Fig. 6. This is because the nontargeted effect was fully induced for such high-dose irradiations, i.e. *S*_NT_ = 1 – κ, irrespective of the radiation type. Thus, its consideration caused little impact on the RBE calculation. On the other hand, the RBE-weighted doses significantly increased with an increase of *x*, which is the ratio of cells that overexpress Bcl-2. This result indicates the importance of the consideration of the Bcl-2 effect in the treatment planning of heavy-ion therapy. This tendency is attributed to the fact that the SF for carbon-ion beams does not change very much due to the consideration of the Bcl-2 effect, but the corresponding iso-survival photon doses became larger owing to the consideration.

The RBE at the center of SOBP was approximately 2.2 and 3.4 for *x* = 0 and 1, respectively. These values were higher than the corresponding data calculated by the treatment planning system for Heavy Ion Medical Accelerator in Chiba (HIMAC) [45]. However, the parameters used in each model were determined to fit the experimentally determined SF of a certain cell line. Thus, determination of the model parameters for various cell lines is needed before quantitatively analyzing the overall influence of Bcl-2 overexpression on the therapeutic efficacy of heavy-ion therapy.

### Conclusions

We have here developed a model assembly for estimating cell surviving fraction related to both targeted and nontargeted effects on the basis of the microdosimetric probability densities for domains and cell nuclei. Radioresistance caused by Bcl-2 overexpression was also considered by introducing the concept of the adaptive response. The model assembly reproduced the experimentally determined SF of Bcl-2 cells and Neo cells irradiated with microbeam or broadbeam very well. However, large uncertainty still remains in the fitting parameters as well as the model concept due to the lack of experimental data.

The consideration of the Bcl-2 effect results in the increase of the RBE-weighted doses for both mono-energetic and SOBP carbon-ion beams, indicating that the developed model assembly can play an important role in the treatment planning for heavy-ion therapy. On the other hand, the consideration of the nontargeted effect is not so important in the estimate of the RBE-weighted doses for heavy-ion therapy, but it is expected to be beneficial to the treatment planning for brachytherapy and boron neutron capture therapy where irradiated and non-irradiated cells coexist. For more quantitative analyses, further studies are necessary for precisely determining the model parameters for various cell lines.
